# Thin Slice Sampling of Video Footage for Mother/Child Interaction: Application to Single Cases

**DOI:** 10.1007/s10862-012-9282-9

**Published:** 2012-04-28

**Authors:** Deborah M. James, Meghana B. Wadnerkar, Christa Lam-Cassettari, Sujin Kang, Anna L. Telling

**Affiliations:** 1 Health, Community and Education Studies, Coach Lane Campus, Northumbria University, Coach Lane, Benton, Newcastle upon Tyne, NE7 7XA UK; 2 NIHR Biomedical Research Unit in Hearing, University of Nottingham, Nottingham, UK

**Keywords:** Video, Mother child interaction, Microanalysis, Contingency, Sequential Analysis, Thin slice sampling

## Abstract

The purpose was to test the reliability of short samples of parent/child interaction for use in single-subject research. Four variable pairs of mother/child behaviour were coded for seven mother/child play sessions. Each session lasted 20 min and 18 min of the session was behaviourally coded using frame-by-frame analysis. The co-occurrence of the mother/child behaviours within a given time window was computed and an odds ratio was calculated for the co-occurrence of the targeted behaviours. The play session was divided into shorter segments (3, 6 and 9 min) and odds ratios of the variable pairs from the shorter segments were compared to the odds ratios from the entire session. Segments of 3 and 6 min did not yield the same pattern of results as the entire session. In single-subject research, evidence of the reliability of the time segment for behavioural coding should be reported in the methods section of original research manuscripts.

Behavioural coding of live or recorded observations is often used as a tool to capture the intricacies of human interaction in a variety of fields for experimental and clinical purposes. It is an accepted practice to code a proportion of the observations or ‘thin-slices’ of interaction as a representation of the whole interaction. The term ‘thin slicing’ was coined by Ambady and Rosenthal (1992, 1993). They were interested in exploring the phenomena that in real-life situations people could make accurate assessments of other people’s personality characteristics based on only a few moments of observing or engaging in interpersonal interaction. Their meta-analysis (Ambady and Rosenthal 1992) showed that the ability to classify people according to closed set character traits was not affected by the length of the sample of interaction that was observed and predictions based on samples of less than 5 min in duration yielded significant effect sizes based on the accuracy of classification of character.

Thin-slicing can be analogues to brief functional assessments that are often used in psychiatry. Brief assessments are known to be reliable, but the results they provide can be limited to behaviours that occur more frequently (Derby et al. 1992) with some studies showing lower accuracy from brief assessments when compared to extended assessments (Roger et al. 1990). Reliability of behavioural observation could also be related to the frequency of occurrence of behaviour so the duration of the observation window could have an impact on reliability. We conducted a review of research studies that used micro-analytic methods to code interaction behaviour between parents and children. There were 38 published studies that matched our search criteria in the previous 20 years. Eighteen of those studies used observation windows of 5 min or less for coding and only 12 studies contained a justification for the duration of the observation window (see Table 1). Of these studies, the monograph of Jaffe and colleagues (2001) provided the highest degree of detail and they cite the meta-analysis by Ambady and Rosenthal (1992) as the basis for their justification of ‘thin slice’ sampling. This raises the question of how thin the slice of interaction needs to be in order to accurately represent the parent/child interaction. This is especially pertinent in the context of mother/child play where the frequency of indicative behaviour is likely to vary widely with context. Prior research suggests that data based on samples of parent/child interaction selected from the beginning of a play session is more representative than samples from the middle or end of the play session (e.g., Beebe et al. 2010; Friedman et al. 2010), but these conclusions were drawn from largely impressionistic analysis rather than comparison of behavioral coding from different portions of the play session.

**Table 1:** Micro-analytic studies of parent–child interaction

Authors	Year	Outcomes measured	Time slice	Justification for time slice duration
Beebe, et al.,	2007	Mother–infant self- and interactive contingencies	First 2.5 usable mins	Followed previous research
Beebe, et al.,	2010	Mother–infant interaction at 4 months	First 2.5 usable mins	Followed previous research
Bornstein	2002	Continuous versus time-sampled observations of mother–infant interaction	Whole video versus time-sampled 15, 30 and 45 s slices	Followed previous research
Feldman	2006	Mother–infant social synchrony in face-to-face play	Middle 3 × 1 min	Followed their own previous research
Feldman, Greenbaum & Yirmiya	1999	Effect of affect-synchrony on infant self-control	First 3 min, (the initial minute was excluded as an adjustment period)	Pilot study, *n* = 5
Feldman, Greenbaum, Yirmiya & Mayes	1996	Relationship between interactive rhythms and cognitive development	First 3 min, (the initial minute was excluded as an adjustment period)	Pilot study, *n* = 5
Friedman, Beebe, Jaffe, Ross & Triggs	2010	Microanalysis of infant vocal affect with depressed mothers	First usable 2.5 min	Followed previous research
Jaffe	2001	Rhythmic coupling and bi-directional coordination in mother–infant interaction	Time-sampled 12 × 1 min	Followed previous research
Ray & Tickle-Degnan	2004	Coding scales for video-taped mother-infant interactions	Time-sampled 5 × 60s and 5 × 30 s	Pilot study
Rodriguez et al.,	2010	Effect of Healthy Families New York in promoting parenting competency	*M* duration of task interval	To suit study design and aims
Vigil	2002	Cultural variation in infant attention regulation	Middle 5 min	Previous research and to suit study design and aims
Weiss, Wilson, Hertenstein & Campos	2000	Tactile contact during mother–infant interactions	Middle + final 5 min	Followed previous research

In the wider arena of behavioural research detailed methodology on the type of sampling has been reported. In a comparison of continuous sampling versus timed-sampling techniques, Bornstein (2002) showed that the patterns of the data derived from the two alternative methods were identical, or identical enough to render any difference to fall within acceptable limits for researchers. The two methods in Bornstein’s study require different amounts of resource. Time-sampling requires less resource than continuous sampling. The resource differential between coding methods highlights the way that the practicalities required for micro-analytic behavioural coding interacts with the choice of sampling methodology. In clinical settings the use of video footage for assessment purposes is increasing (Lord et al. 1989, 2000; Tait et al. 2001) and at the same time it is likely that the resources available for micro analysis of that footage is decreasing. Clinical scientists designing new applications using micro-analysis of behavioural data might be inclined towards thin-slice coding since it is quick and there is no evidence to question its reliability from experimental research.

The interaction between parents and children carries critical determinants for infant socio-cognitive development (Belsky and Fearon 2002; Bernier et al. 2001; Biringen et al. 2005; Bornstein 1985; Feldman and Eidelman 2009; Trevarthen and Aitken 2001). Maternal behaviours such as the expression of affect (Moore et al. 1997), eye-gaze towards the child (Stern 1983), touch (Tronick 1995), and vocalisation (Hsu and Lavelli 2005) have been found to be predictive of a range of infant outcomes (Feldman et al. 1999, 2004b; Gable and Isabella 1992; Hsu and Fogel 2001; Hsu et al. 2000; Hsu and Lavelli 2005; Moore et al. 1997; Murray and Hornbaker 1997; Treyvaud et al. 2009). Interactional parental behaviour has been found to vary according to culture (Feldman et al. 2006; Hsu and Lavelli 2005), presence of maternal postnatal depression (Milgrom et al. 2004) and the status of the sibling/s within the family unit (Feldman et al. 2004a; Moore et al. 1997). Research has shown that it is not just the type and frequency of the parents’ early interactional behaviour that is important, but the contingency of parental behaviours is also crucial for the child’s development (Murray and Trevarthen 1985).

Contingency of parental behaviour can be understood as the sequence and timing of parent’s behaviours within which the parent responds to the child’s cues. It is considered to be a property of a wider parenting construct referred to as maternal responsiveness or maternal sensitivity (Shin et al. 2006). In the same way that interactional behaviour varies with context, contingency of parental behaviour is also subject to variation. In experimental research depression in mothers has been found to influence the contingency of the mother’s responses during interaction exchanges with infants. The measurement of the contingency of behaviours during dyadic interaction is time intensive, but its current status within theoretical models of development (Jaffe et al. 2001) and its use in the design and evaluation of intervention programmes for parents (Rodriguez et al. 2010) means that the coding of contingency during interaction will continue for some time to come.

In the implementation of an exploratory phase 1 clinical trial of a parent intervention programme in the context of childhood hearing impairment, we chose to use a measure of parental contingency to evaluate change before and after the intervention. This led us to question, how much parent/child interaction we needed to code to get a reliable and representative measurement of contingency. The reliability of the data was particularly important since we planned to use the measure to assess change over time in single families. The analogous application of Ambady and Rosenthal’s study to the field of developmental psychology might be articulated as such; parental contingency is an observable and measurable expressed behaviour; could brief observations of this behaviour be used to accurately judge the trait of parental responsiveness.

In the larger study for which we were defining our methodology, our intention was to test an experimental hypothesis which stated that the parent intervention in the clinical trial would enhance parental responsiveness. This hypothesis drove the need to develop a robust measure of parental contingency which examined the sequential occurrence of the parents’ behaviour in response to the child’s. The dependent variable was for use in a phase 1 clinical trial with single-subject design and there was a need to test the reliability of the measurement for use with single cases. It was not enough to adopt methods that had been derived from pooled data from experimental research. So there remained a question as to whether the coding of a thin slice (5 min or less) of behaviour would yield results similar to that provided from a complete play session of 20 min duration. The practical question of how much data we needed to code to get a reliable result meant that we compared three partial segments that differed in duration, one time slice that was below 5 min duration (3 min segments—referred to as *short duration*), one time slice that was above 5 min duration (6 min—referred to as *medium duration*) and one time slice that represented half of the entire play session (9 min—referred to as *long duration*). The main experimental hypothesis of the study is that short slices of behavioural data (less than 5 min in duration) taken from naturalistic free play between mothers and children is not as reliable as longer slices of behavioural data (medium duration and long duration). A secondary experimental hypothesis of the study is that the frequency of behaviour, based on a count from the population of the entire free-play session, interacts with the reliability of data from the differing time samples. Low frequency contingency pairs will be less reliable in all the samples, but particularly in the short samples compared to higher frequency contingency pairs. A final experimental hypothesis of the study is that results based on the samples taken from the beginning of the entire play session will be more reliable than samples taken at the end of the play session. This pattern will be observed for all the sample durations (short, medium and long).

The analysis will be based on assessment of the likelihood ratios of behaviours of mother and child co-occuring within a specific time window on both case-by-case basis and grouped data. This study is based on a large scale intervention study looking at mother–child interaction and child speech development where the child is Deaf/deaf/hard of hearing. As part of a larger assessment session testing a range of psychological and acoustic speech measures associated with child development and parenting, a mother and her Deaf/deaf/hard of hearing child were video recorded while engaged in a naturalistic free play interaction.

## Method

### Participants

This study uses data from four mother–child dyads who were taking part in the aforementioned intervention study. There were two boys (ages 3;10 and 6;10 years) and two girls (ages 5;3 and 1;11 years). All mothers were hearing and all children were congenitally profoundly deaf. Two children had bilateral cochlear implants and were pre-lingual. One child (girl 5;3 years) had bilateral cochlear implants, and had some level of speech. One child had a unilateral cochlear implant and was pre-lingual with severe learning difficulties (boy aged 6;10 years).

For the two girls, their father and sibling were also present in the room to keep the sessions as representative of the family as possible. However, the presence of additional interactive partners made the behavioural coding more complex than traditional face-to-face microanalytic studies. For coding purposes only interaction between the mother and her deaf child was coded. Two families had one pre-intervention visit each and two families had two visits each (pre and post intervention baseline) following the same procedures (family 1—one visit, family 2—three visits, family 3—two visits and family 4—one visit). Since the focus of this study is the methodology of choosing time segments for behavioural analysis, the intervention status of the families was not considered as part of the analysis. This gave a total of seven video cases for analyses in this paper.

Informed, signed consent was obtained from the parent/caregiver prior to starting the study. The study has been reviewed and approved by the Derbyshire Research Ethics Committee and Nottingham University Hospitals NHS Trust Research and Development department. All families were reported to be in good health at the time of filming.

### Recording and Editing

Families were video recorded during an unstructured 1 free play interaction in the family lab at NIHR National Biomedical Research Unit in Hearing. The lab resembles a family lounge and consists of two lounges, a child’s desk and chair, and a variety of toys to support play sessions. To minimise the obtrusiveness of the equipment, recordings were taken via three wall mounted hard-drive video cameras that were disguised with fluffy toys so that only the lens was showing. The mothers were instructed to play and spend time with their child as they normally would at home, using any of the toys available for 20–25 min. They were allowed to take a break if required. Following the test session, each camera file was converted into a 20 min movie in MPEG4 format to ensure each camera angle was 25 frames per second.

### Behavioural Coding

Video recordings were coded as a continuous variable in INTERACT software (Mangold 2008) to extract the frequency, onset, offset and duration of each mother–child dyads’ gaze and verbal behaviours. Videos were coded frame-by-frame in the format 25 frames per second. Coding was shared by two coders. Each mother–child dyad was coded independently for each behaviour, giving four codes per dyad: eye gaze of the child towards the mother coded as ‘child look’; child’s vocalisations coded as ‘child verbal’; eye gaze of the mother towards the child coded as ‘mother look’ and mother’s speech coded as ‘mother verbal’. These behaviours were selected in line with the hypotheses for the larger study which are centred on maternal attunement as measured by eye gaze and speech. The larger study seeks to understand the effect of an intervention on mother’s contingent responses to the child. Hence maternal behaviours are defined as ‘Target’ behaviours and child behaviours are defined as ‘Given’ behaviours (Bakeman and Quera 2011b). A third coder blind coded 20% of randomly selected footage from each 18 min file for each of the seven cases to calculate inter rater agreement. This equated to 3.6 continuous minutes per video. There was a good agreement between the coders as indicated by the mean κ = 0.85 for overall child behaviours (child look κ = 0.96 and child verbal κ = 0.92) and for overall mother behaviours κ = 0.76 (mother look κ = 0.97 and mother verbal κ = 0.95).

To account for the time taken by the participants to settle in the activity the first and last 1 min was discounted from the 20 min free play video while coding, thus leaving 18 min of free play to code. In the first instance the whole 18 min was coded for each of the seven videos. Each 18 min code file was then divided into six slices that were 3 min in duration (first 3 min, followed by the second 3 min etc.). In addition the 18 min of coding was divided into two segments of 9 min duration and three segment of 6 min duration. Each coded segment is referred to as a dataset in the analysis and results section.

### Data Extraction

The coding software INTERACT stores data sets as tab-delimited text files (.ACT files). The .ACT files were extracted in ActSds using 1 s time units (Bakeman and Quera 2009) and converted into Sequential Data Interchange Standard (SDS), a format used for sequential data analysis (Bakeman and Quera 1992, 2008). These .SDS files were opened and modified for time-event sequential analysis in Generalized Sequential Querier software (Bakeman and Quera 1995, 2008, 2011a). If required, this data can then be transferred into any standard statistical programme for further analysis or modelling. See Bakeman and Quera (2011b) for details on sequential analysis.

### Data Analyses

In the first instance, observed raw frequencies were computed on the raw data for the child looking and verbal behaviours and mother’s looking and verbal behaviours for each of the time slices (i.e., 18 min, 9 min, 6 min and 3 min) for each of the seven video cases in GSEQ.

In order to analyse whether the mother’s looking and verbal behaviours (Target behaviours) were contingent within 3 s of the child’s looking and vocalisations (Given behaviours), the WINDOW command in GSEQ was used to define four new codes: One coded the onset (first unit) second of the Given behaviour and the following 2 s as a Given window for the child’s looking and vocalisations separately, while the other two coded just the onset of the mother’s looking and verbal behaviours separately (Bakeman and Quera 2011b). The four behaviours with modified time windows provided the opportunity to perform sequential analysis in GSEQ and were tallied in a 2 × 2 table for each behaviour pair (child looking with mother looking, child looking with mother verbal, child verbal with mother looking, and child verbal with mother verbal) for each of the time slices (i.e., 18 min, 9 min, 6 min and 3 min) for each of the seven video cases. Contingency indices (Joint Frequency, and Logg odds ratio) were computed for these (see Chorney et al. 2010 for further explanation of methodology for sequential analysis).

Results from the raw data and sequential analysis were transferred into SPSS (SPSS 2007) and Microsoft Excel (2007). Results were analysed at a descriptive level using mean and standard errors of raw data frequencies, joint frequencies and contingency indexes (Logg odds) for each behaviour pair across the seven cases to examine whether the time slices provided different results from that derived from the whole 18 min free play session for each behaviour pair. Due to limited number of cases (seven cases) a modelling approach such as Repeated Measures ANOVA or Generalized Linear Mixed Models with Logg odds (since distribution of data with odds ratio was skewed) could not be considered. In any case, our focus on the reliability for individual case data meant that we were largely concerned with pattern analysis on a case-by-case basis.

## Results

Contingencies were computed of mother’s behaviour in response to the child’s behaviour. Mother behaviours were re-coded in GSEQ to follow the child behaviours within a 3 s time window. Due to the small sample size and odds ratio being skewed, the Logg odds ratio was computed as the contingency indices. The odds ratio is a measure of effect size and gives the direction of association. The Logg odds ratio is the natural logarithm of the odds ratio and varies from negative infinity to positive infinity, with zero as no effect. A Logg odds ratio of greater than zero indicates that the *target* behaviour is more likely in the presence of the *given* behaviour and a value smaller than zero indicates the opposite (Bakeman and Quera 2011b). For example the parent is more likely to speak (target) within the three second time window after the child has made a verbalisation (given). The data from all seven cases were analysed using the Logg odds ratio and are presented as single cases and as grouped data.

The data for the single cases are presented in Table 2. To assess the reliability of the short samples all results for each variable pair that differed in Logg odds ratio across the zero boundary were identified. Any value in the opposite direction of the Logg odds was defined as a discrepancy. If the Logg odds value was positive, based on analysis from the entire play session for a particular dyad, then a negative value was considered as discrepant and vice versa. These discrepancies are highlighted in Table 2. In all seven cases the shorter segments (3 or 6 min) yielded Logg odds ratios that differed from the Logg odds derived from the whole play session across the zero boundary. Values for the longer duration of 9 min, especially for the first 9 min, closely matched values for 18 min.

**Table 2:**
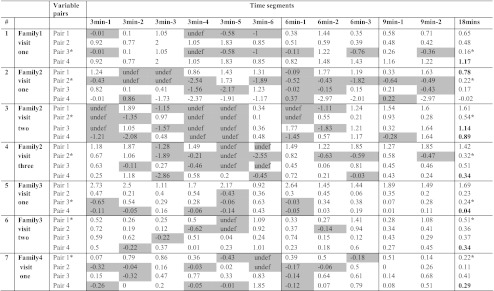
Logg odds ratio for the case by case data for each time slice (3 min, 6 min, 9 min) and entire play session (18 min). Pair1: Child look & mum look; Pair 2: Child look & mum verbal; Pair 3: Child verbal & mum look; Pair 4: Child verbal & mum verbal. Undef: when there were no co-occurrences of contingent child and mother behaviours the Logg odds could not be computed and are termed as undefined. Low frequency variable pairs are marked *; High frequency variable pairs are marked in bold in the final column

The second research question concerned the reliability of low frequency pairs. Low frequency contingency pairs were identified using descriptive statistics from the data from the entire play session. The lowest frequency pair for each dyad is marked with an asterisk in Table 2. The data suggest that there was a higher likelihood of the low frequency contingency pair being uncomputable and this is especially the case in the shorter segements (labelled as ‘undef’). However, other pairs, that were not low in frequency were also left undefined in the computer analysis, again, most notably in the short segments.

The final question concerned the time of the play session when the segment was sampled (beginning of session, middle or end). The data from the single case analysis is inconclusive in this regard. There is no evident difference in the degree of discrepancy when the segments were sampled from the beginning, middle or end of the play session from the data in Table 2.

We set out to test the primary research question with the grouped data from the seven dyads. This was done by comparing the mean of the Logg odds ratios from the short segments with the mean and standard deviation of the entire play session. *Z-*scores were computed for the Logg odds derived for each variable pair from each of the short segments. The *z-*score is an indication of the probability of obtaining a score within a standard population. In this study, the standard population is the entire play session. The degree of variability within the standard population affects *z-*scores. In statistical terms, the distribution of the standard normal population is characterised by the mean and standard deviation. In a normal distribution 95% of the population lie within ±1.96 *SD*s from the mean. A *z-*score of 2 or more (either plus or minus) can be considered to lie outside of the variance of the standard population since only 5% of the scores from the standard population would have fallen at that observed score. Therefore, for our analysis, *z-*scores of 2 or more were categorised as falling outside the normal distribution of the standard population.

**Fig. 1:**
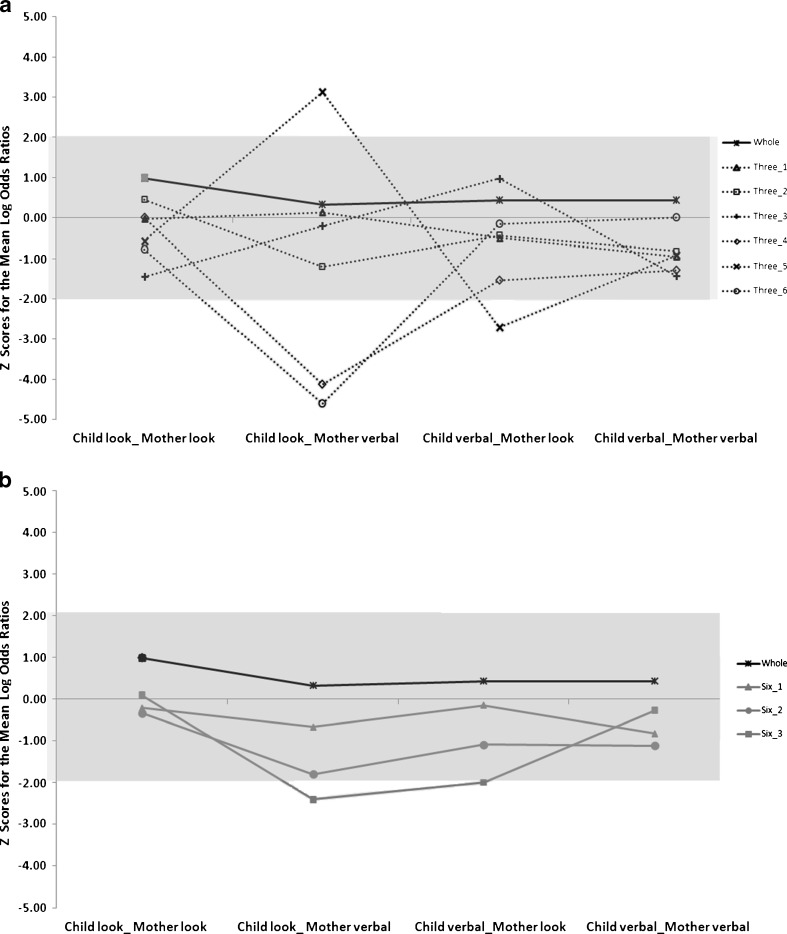

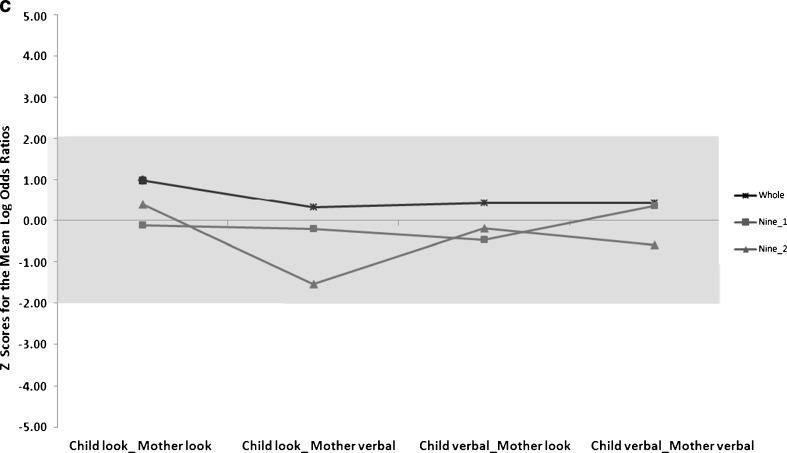
**a** *Z-score* calculated on the mean and standard deviation of the Logg odds ratios from the entire play session (18 min), compared to each of the 3 min segments. **b** *Z-score* calculated on the mean and standard deviation of the Logg odds ratios from the entire play session (18 min), compared to each of the 6 min segments. **c** *Z-score* calculated on the mean and standard deviation of the Logg odds ratios from the entire play session (18 min), compared to each of the 9 min segments

*Z-*scores for each of the shorter segments were derived for each of the contingency pairs. This data are in Fig. 1. The graphs are presented separately for each time slice i.e. all parts of 3 min (Fig. 1a), all parts of 6 min (Fig. 1b) and both parts of 9 min (Fig. 1c). Here the horizontal axis at 0 represents the benchmark as derived from the mean and standard deviation from the 18 min play session. The lines represent the *z-scores* (standard scores) for each of the shorter segments across all four contingency pairs. Lines that are closer to zero deviate less from the Logg odds computed from the entire play session. The contingency pairs in the 9 min segments are within one *z*-score of the entire play session. Some of the *z*-scores of the contingency pairs from the 6 min and 3 min segments fall outside one standard deviation of the mean of the entire play session. Some of the *z*-scores from the shorter segments (6 min and 3 min) fall well below two standard deviations (usually in a negative direction, but not always) thus indicating wide variation from the findings of the entire play session.

These data suggest that for some contingency pairs (child look_mother verbal) there was particularly wide variability when data from the short segments are compared to each other. In three of the seven cases, this contingency pair was the pair that occurred least frequently in the entire play session. With regard to the question about the position in the play session that data is sampled from, there is an indication, from observing the data in Fig. 1 that the first 9 min sampled yielded more reliable results than the 9 min segment sampled from the end of the play session.

## Discussion

The findings from the single subject and group analysis presented here show that in single subject research, short time slices (3 min and 6 min) produced discrepant findings compared to the findings from the whole population (18 min). The results suggest that coding based on half the entire play session yielded results that were neither discrepant nor widely varying when compared to the findings derived from the entire play session. Given the centrality of contingency to the hypothesis in the larger study for which our methods were being defined, the discrepancy, and the direction of that discrepancy derived from the short time slices provided significant methodological justification for the design of our analysis of a larger study. If we had used 3 min segments to code data then our conclusions would have differed considerably from the findings based on an entire play session. For example, we would have often concluded that a mother was unlikely to show verbally contingent behaviour on her child’s vocal behaviour where, in fact, this was not the case. Therefore, the experimental hypothesis is supported.

For the wider study for which we were developing our methodology the findings from this small set of cases is enough to generate a well rationalised approach to the sample selection in our single subject design. Clearly, the sample size to be selected is 9 min in duration and the beginning 9 min will be used since this was the only sample that yielded consistently non discrepant results from that of the population. If we were inclined to make a judgement about the parenting style of the parent according to level of responsiveness to the child, based on the shorter 3 min or 6 min observations, we would have classified her differently from the classification that we would have made based on a longer observation. Thus, from these data, in this context, the applicability of Ambady and Rosenthal’s thesis, which provides a rationale for thin-slice sampling, was not supported.

A limitation of this study is the small sample size. Whilst this does not impact on the applicability of the findings from this study to our own single case study research or to others who might be using micro-analytic behavioural coding in single subject research, these findings do not have any implications for research adopting group designs. The findings presented here cannot be used by other research groups to provide a rationale for the specific window duration for sample selection of behavioural data. Participant numbers, the number of variable pairs coded, and the frequency of the measured behaviours will all have an impact on the power to detect the significant patterns within behavioural data. The value of this research report lies in the attention that it draws to the need for researchers to develop and test their methodology for window duration decisions for clinical and experimental research using behavioural coding.

In the review of recent research in the field we were surprised to find how often no rationale on the sampling choices was provided in the study methodology. Our response to this has been to make explicit our own steps to producing robust micro-analytic methods for single-case studies. We think this study provides new knowledge which is relevant to those involved in the design or use of video footage in the applied contexts of pediatric family assessment and diagnosis. Given the constraints on resources in clinic and research, clinical scientists designing new applications using micro-analysis might be a) inclined towards thin-slice coding due resource considerations and b) draw the conclusion that thin slices of behavioural coding gives reliable indicator of a state or trait in single cases based on the group findings in experimental research. Given the potential use of clinical applications using video, such as in assessment and diagnoses and design of therapeutic interventions we urge caution in the application of thin-slice methodology and underline what Bornstein suggests, that every researcher should choose the acceptable limits of unreliability for their study. For our laboratory, making a choice based on previous research was not possible given the way in which we judged the applicability of the prior gold standard evidence on thin-slice coding to our research. Our aim in articulating our choice making process, including the results of our test is to model what Bornstein (2002) advocated and to show the benefit in questioning the basic premise that underpins methodological choices in this field.

## Footnotes

1 Unlike a face-to-face interaction or structured play the mother–child pair is free to choose their play activity and move about in the room.
